# A cardiac-rehab home-based mHealth program to improve physical activity in patients with coronary artery disease: a randomized controlled trial

**DOI:** 10.1007/s12471-026-02039-5

**Published:** 2026-04-09

**Authors:** Sophie H. Kroesen, Thijs Vonk, Malou A. H. Nuijten, Erwin S. Zegers, Maria T. E. Hopman, Esmée A. Bakker, Thijs M. H. Eijsvogels

**Affiliations:** 1 Department of Medical BioSciences, Radboud University Medical Center, route 928, Nijmegen, The Netherlands; 2 Department of Cardiology, Canisius Wilhelmina Ziekenhuis, Nijmegen, The Netherlands; 3 Department of Primary and Community Care, Radboud University Medical Centre, Nijmegen, The Netherlands; 4 Department of Physical Education and Sports, Faculty of Sport Sciences, Sport and Health University Research Institute (iMUDS), University of Granada, Granada, Spain

**Keywords:** Cardiac rehabilitation, Prevention, Cardiovascular disease, Exercise, E‑Health

## Abstract

**Purpose:**

Contemporary cardiac rehabilitation (CR) has a moderate effect on physical activity (PA), whereas novel technologies offer promise for enhancing PA levels. Therefore, we assessed the effect of a home-based smartphone training program in addition to center-based CR on PA levels in patients with coronary artery disease (CAD).

**Methods:**

CAD patients participating in CR were included in this randomized controlled trial (1:1, stratified for index diagnosis). The control group received usual care CR, whereas the intervention group additionally received a 6-week remote smartphone program. The primary outcome was the change in accelerometer-derived moderate-to-vigorous PA (MVPA) from baseline to post-CR. Secondary outcomes included changes in light intensity PA, step count, sedentary time, functional parameters, quality of life, and cardiac anxiety. A baseline-adjusted linear mixed model was used.

**Results:**

Participants (16% female, intervention *n* = 44, control *n* = 49) were 63 [56–69] years old and had a baseline MVPA of 1.0 (95% Confidence interval (CI): 0.9; 1.1) h/day. Changes in MVPA did not differ between the intervention (0.1 (95% CI: −0.0; 0.2) h/day) and control group post-CR (0.1 (95% CI: −0.0; 0.2) h/day, *p*_-interaction_ = 0.75). Also, no differences between the groups were observed for light intensity PA (0.5 (95% CI: 0.2; 0.8) *versus* 0.4 (95% CI: 0.1; 0.8) h/day, *p*_-interaction_ = 0.79). Similarly, changes in other secondary outcomes did not differ among groups.

**Conclusions:**

A smartphone training program on top of the usual CR did not yield additional benefits. A more elaborate mHealth intervention seems needed to change PA during CR in active patients with CAD.

**Supplementary Information:**

The online version of this article (10.1007/s12471-026-02039-5) contains supplementary material, which is available to authorized users.

## What’s new?

- Novel mHealth strategies are increasingly used in cardiac rehabilitation to improve outcomes, but the optimal delivery method, timing and technology are unknown.
- This leads to the question what the minimal amount of technology and guidance is to achieve physical activity changes.
- Our randomized controlled trial in 93 participants showed that a 6-week smartphone delivered training program in addition to current cardiac rehabilitation does not increase objectively measured physical activity levels.
- A more elaborate mHealth approach including several behaviour change techniques and repetitive bi-directional contact seems necessary to augment the effects of cardiac rehabilitation on physical activity.

## Introduction

Cardiovascular diseases (CVDs) are the leading cause of morbidity and mortality worldwide [1]. To reduce the burden of CVD, secondary prevention strategies are essential, and exercise-based cardiac rehabilitation (CR) is strongly recommended by international guidelines [2] as it enhances cardiac function, physical fitness, and CVD risk factors [3]. Nevertheless, adherence to center-based CR programs is suboptimal, partially caused by logistical barriers and the lack of a personalized approach [4]. Furthermore, post-CR changes in physical activity (PA) are small-to-moderate at best (0 to +0.3 h/day) [5, 6], highlighting the poor translation of CR into the home environment.

Home-based CR programs using novel mHealth applications are shown to be as effective as centre-based CR [7, 8], where home-based exercise training with remote coaching can support long-term PA by developing self-management skills [7]. However, mHealth add-ons to CR is a relatively new strategy and the optimal delivery method, format, and technology remain unknown to date [8]. This raises the question if a hybrid approach can be more effective compared to center-based CR alone [8]. Therefore, the Cardiac RehApp randomized controlled trial assessed the effect of a home-based smartphone training program in addition to center-based CR on PA levels in patients with coronary artery disease. We hypothesize that the combined approach of home-based training and center-based CR would result in greater improvements in PA levels compared to center-based CR only.

## Methods

### Study design

We performed a randomized controlled trial the rationale and design of which, including the sample size and power calculation, have previously been described in detail [9] and are summarized in the Electronic Supplementary Material [ESM] File S1. Patients enrolled to CR at Canisius Wilhelmina Ziekenhuis (Nijmegen, the Netherlands) were eligible if they were > 18 years, had a diagnosis of coronary artery disease, and were able to understand and perform the study procedures. Exclusion criteria included inability to use the smartphone application, contraindications, or severe orthopedic problems that restrict PA. Informed consent was obtained from all patients prior to participation, and the Cardiac RehApp trial protocol adhered to the ethical guidelines of the 1975 Declaration of Helsinki as reflected in a priori approval by the Medical Ethics Committee of the Radboud University Medical Center (NL72182.091.19). The CONSORT checklist is available in ESM Table S1.

Inclusion and baseline measurements took place prior to the CR program. Thereafter, they were randomly allocated (1:1) to the intervention or control group using a computerized algorithm with allocation concealment and random block sizes (4-6) stratified for index diagnosis (acute events *versus* elective procedures). Directly after the 6‑week program, post-CR measurements were performed. Only outcome analyses were blinded.

### Cardiac rehabilitation and intervention

All patients received usual care consisting of a ~ 6-week comprehensive center-based CR program. The program included consultations focusing on lifestyle improvement, medication adherence and psychosocial well-being. Additionally, patients participated in a supervised exercise program and, if appropriate, a dietary module, psycho-educative prevention module and psychological module.

Participants in the intervention group received a 6-week smartphone training program alongside usual care CR. The participants were instructed to perform daily physical activities in their home situation using the Virtual Training mHealth smartphone application (Welfaster ApS, Denmark) [9]. The personalised home-based program was configured by the research team to ensure safety and feasibility during the trial. The application contained different training programs, including strength and aerobic exercises (e.g., squats, walking, cycling) and synchronous instructions by video, text, and audio. At baseline, the researcher and participant set individual goals based on preferences (e.g., biking or walking) and physical status (i.e., physical limitations, previous exercise experience, age), which were converted into a personalized home-based training program by the researcher. The smartphone program stimulated daily PA through goal-setting, personalised exercise prescriptions, and automated reminders in the home environment. Progression and feasibility were monitored via in-app messaging, and participants could contact the research team through the interactive platform when needed. These elements reflect common behaviour-change techniques such as action planning, prompting, and feedback, which are known to support increases in PA levels [10].

### Outcome measures

All outcomes were measured at baseline and Post-CR. PA patterns were objectively measured during 8 days with a validated thigh-worn accelerometer (ActivPAL micro, PAL technologies, Glasgow, United Kingdom) [11] and analyzed by a modified version of the script of Winkler et al. [12]. Moderate-to-vigorous PA (MVPA), light intensity PA (LIPA), and sedentary time were expressed in h/day. Step count was expressed as steps/day.

The Åstrand test was performed to examine physical fitness [9]. During the test, heart rate was continuously monitored (Polar V800, Kempele, Finland), and the Borg score was reported during the third and sixth minutes. Patients using heart rate-lowering medication (e.g., beta-blockers) followed an adjusted test [9]. Hand grip strength was assessed in the dominant hand using a dynamometer (Jamar, Jackson, MI, USA). Three measurements were performed, and the maximum strength effort (kg) was used for analysis.

Quality of life was measured using the validated HeartQoL questionnaire [13], and cardiac anxiety was measured using the validated Cardiac Anxiety Questionnaire (CAQ) [14].

### Statistical analysis

All statistical analyses were performed using R version 4.2.1 with packages “lme4” and “Lmmstar” (visualization purposes), and “emmeans”. All tests were two-sided, confidence intervals (CI) were at the 95% level, and P‑values < 0.05 were considered statistically significant. Continuous normally distributed data were presented as mean ± standard deviation (SD), continuous not-normally distributed data as median [interquartile range] (IQR) and categorical variables as number (%). All data were visually inspected for normality.

Primary and secondary outcome analyses were performed on a modified intention-to-treat basis, including all participants with at least one primary outcome measurement at baseline or post-CR. A baseline-adjusted (i.e constrained) linear mixed-model analysis was used to handle missing data and to avoid baseline imbalances between treatment arms [15]. Time (categorical) and the interaction time*group were included in the model.

## Results

### Study population

**Fig. 1:**
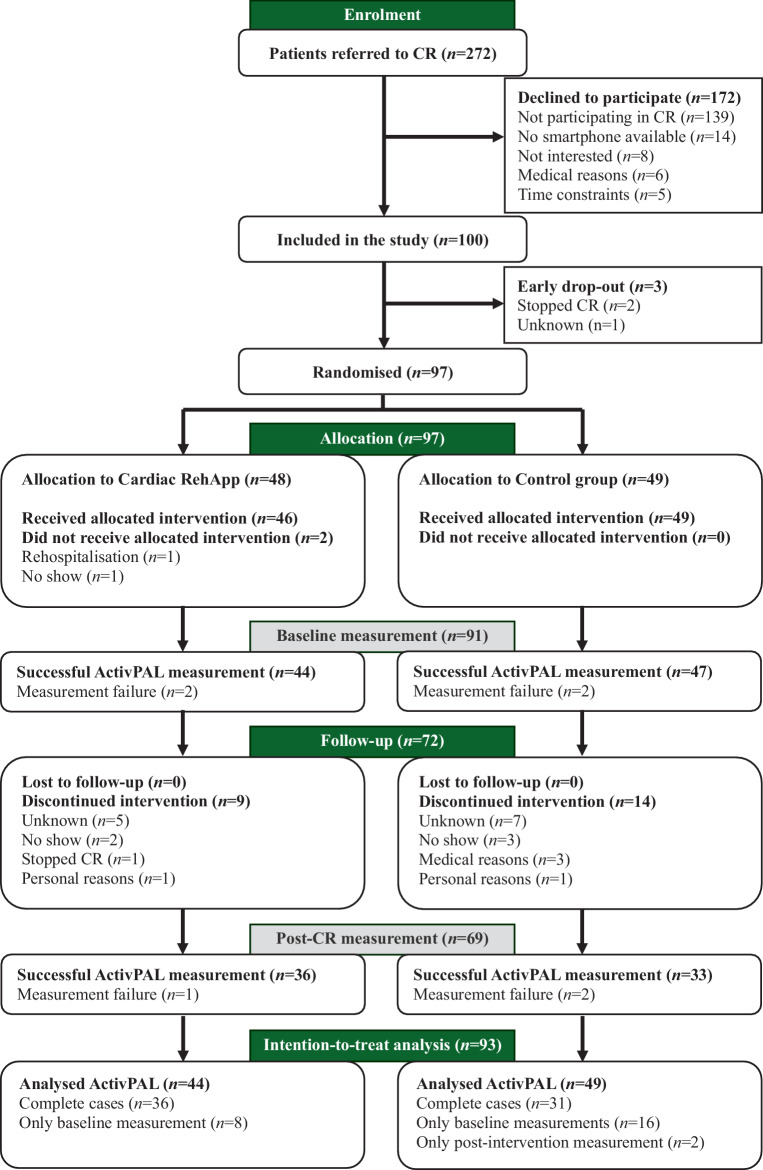
Flowchart of the Cardiac RehApp study

272 patients were approached for study participation between 8 December 2020 and 3 December 2021, of which 100 patients were enrolled (37%). A total of 93 participants (93%, intervention *n* = 44, control *n* = 49) with at least one primary outcome measurement (baseline and/or post-CR) were included in the analytical cohort (Fig. 1). Patients had a median age of 63 [56–69] years, 15 (16%) were female, and 51 patients (55%) were included after an acute hospitalization (Tab. 1). 11 patients (25%) dropped out in the intervention group and 14 (29%) in the control group (Fig. 1). No study-related adverse events were reported.

**Table 1:** Baseline characteristics of the study cohort.

	Total (*n* = 93)	Intervention (*n* = 44)	Control (*n* = 49)
*Patient characteristics*			
Age (years)	63 [56–69]	63 [59–68]	63 [55–70]
Sex (female)	15 (16%)	7 (16%)	8 (16%)
Body mass index (kg/m^2^) (*n* = 84)	28.4 ± 4	28.3 ± 4.5	28.5 ± 4.4
Employed (*n* (%)) (*n* = 78)	46 (59%)*	22 (59%)*	24 (59%)*
*Lifestyle factors* (*n* = 84)*			
Current alcohol drinker (*n* (%))	45 (54%)	23 (56%)	22 (51%)
Current smoker (*n* (%))	9 (11%)	3 (8%)	6 (14%)
*Comorbidities*			
Hypertension (*n* (%))	52 (56%)	28 (64%)	24 (49%)
Dyslipidaemia (*n* (%))	29 (31%)	13 (30%)	16 (33%)
Diabetes mellitus (*n* (%))	12 (13%)	7 (16%)	5 (10%)
Atrial fibrillation (*n* (%))	4 (4%)	3 (7%)	1 (2%)
Heart failure with reduced ejection fraction (*n* (%))	4 (4%)	2 (5%)	2 (4%)
Peripheral artery disease (*n* (%))	3 (3%)	2 (5%)	1 (2%)
Rheumatoid arthritis (*n* (%))	2 (2%)	2 (5%)	0 (0%)
COPD (*n* (%))	5 (5%)	3 (7%)	2 (4%)
*Index CAD diagnosis* (*n* = 84)*			
Non-ST-elevation myocardial infarction (*n* (%))	19 (23%)	8 (20%)	11 (26%)
ST-elevation myocardial infarction (*n* (%))	26 (31%)	11 (30%)	15 (35%)
Stable angina pectoris (*n* (%))	35 (42%)	20 (50%)	15 (35%)
Unstable angina pectoris (*n* (%))	3 (4%)	1 (3%)	2 (5%)
Elective procedure, *n*(%)	42 (45%)	20 (45%)	22 (45%)
*Treatment*			
PCI (*n* (%))	68 (73%)	34 (77%)	34 (69%)
CABG (*n* (%))	13 (14%)	5 (11%)	8 (16%)
Conservative (optimal medical treatment only) (*n* (%))	12 (13%)	5 (11%)	7 (14%)

Data are presented as *n* (%) for categorical variables and as mean (± standard deviation) for normal distributed continuous data or median [interquartile range] for non-normal distributed continuous variables

*CABG* coronary artery bypass grafting, *COPD* chronic obstructive pulmonary disease, *PCI* Percutaneous Coronary Intervention

*Percentages are based on participants without missing values

### Physical activity and sedentary behavior

**Fig. 2:**
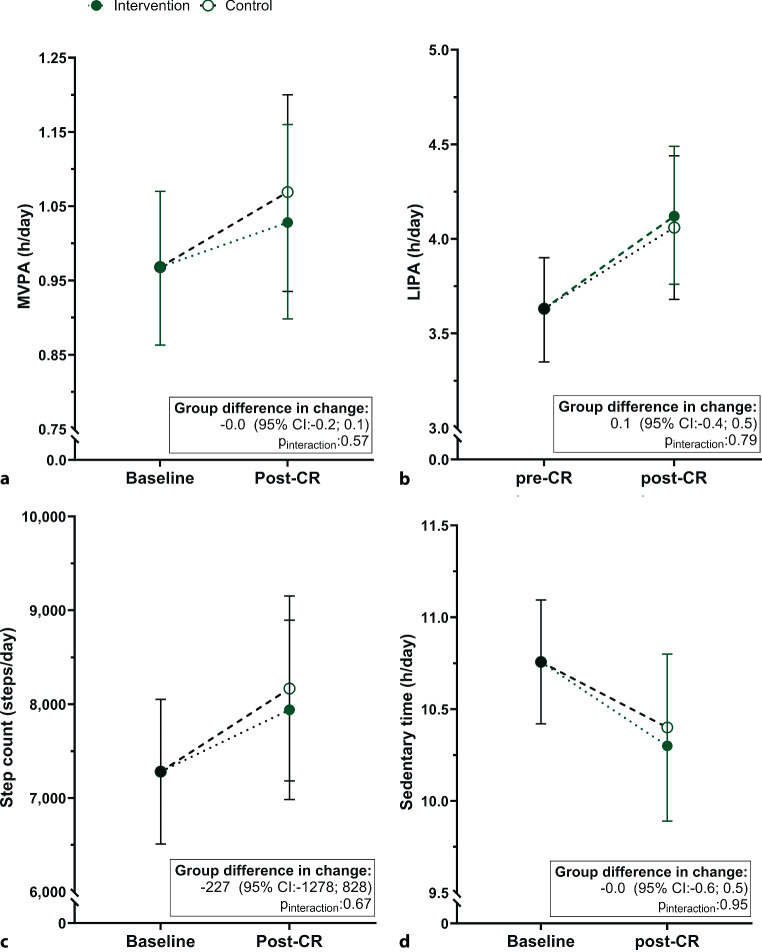
Estimated marginal means of different accelerometry outcomes using a baseline adjusted linear mixed model. The total group (black) at baseline (intervention *n* = 44; control *n* = 47) and the intervention (solid green) and control (open white) group post-cardiac rehabilitation (CR; intervention *n* = 36; control *n* = 33) are depicted. (**a**) Time spent in moderate-to-vigorous physical activity (MVPA). (**b**) Time spent in light intensity physical activity (LIPA). (**c**) Daily number of steps. (**d**) Sedentary time per day. Data are plotted as mean with 95% confidence intervals

Changes in MVPA did not differ between the intervention (0.1 (95% confidence interval (CI): −0.0; 0.2) h/day) and control group post-CR (0.1 (95% CI: −0.0; 0.2) h/day, p_interaction_ = 0.75), Fig. 2a, ESM Table S2). Also no differences in the pre-post changes between the groups were observed for LIPA (0.5 (95% CI: 0.2; 0.8) *versus* 0.4 (95% CI: 0.1; 0.8) h/day), p_interaction_ = 0.79, Fig. 2b), step count (660 (95% CI: −88; 1407) *versus* 887 (95% CI: 88; 1686) steps/day, p_interaction_ = 0.67, Fig. 2c) and sedentary time (−0.4 (95% CI: −0.8; −0.0) *versus *−0.4 (95% CI: −0.8; +0.0) h/day, p_interaction_ = 0.95, Fig. 2d).

### Functional parameters and quality of life

**Fig. 3:**
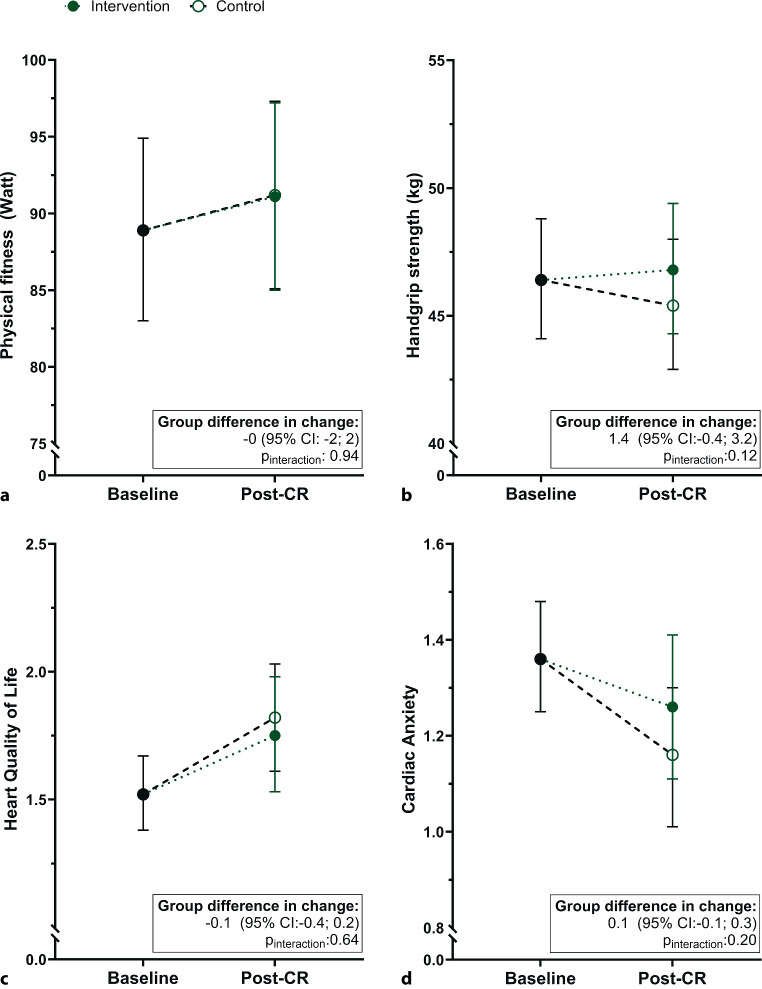
Estimated marginal means of different functional parameters, quality of life and cardiac anxiety using a baseline adjusted linear mixed model. The total group (black) at baseline and the intervention (solid green) and control (open white) group post-cardiac rehabilitation (CR) are depicted. Data are plotted as mean with 95% confidence intervals. (**a**) Physical fitness (baseline: intervention *n* = 42; control *n* = 49. Post-CR: intervention *n* = 35; control *n* = 35), (**b**) Handgrip strength (baseline: intervention *n* = 43; control *n* = 49. Post-CR: intervention *n* = 36; control *n* = 35), (**c**) Heart Quality of Life (baseline: intervention *n* = 39; control *n* = 44. Post-CR: intervention *n* = 28; control *n* = 32), and (**d**) cardiac anxiety (baseline: intervention *n* = 40; control *n* = 44. Post-CR: intervention *n* = 29; control *n* = 34)

Changes in physical fitness (Workload and RPE) and handgrip strength did not differ between the intervention and control group (Fig. 3a, b and ESM Table S2). Similarly, changes in the total or sub scores of quality of life and cardiac anxiety did not differ between groups (Fig. 3c, d and ESMTable S2).

## Discussion

This study determined the effect of a home-based smartphone exercise training program in addition to center-based CR, aiming to increase habitual PA levels in patients with coronary artery disease. We found that the addition of a smartphone training program did not affect the change in habitual PA levels compared to usual CR alone at 6 weeks post-inclusion. Similarly, changes in functional parameters, quality of life, and cardiac anxiety did not differ among groups. These findings suggest that concurrent center-based CR and home-based CR via solely a smartphone exercise program does not yield greater improvements in PA, physical fitness, and subjective outcomes in this stage of rehabilitation than center-based CR only.

### Physical activity

In contrast to our hypothesis, the addition of a smartphone training program to contemporary CR did not yield additional improvements in PA levels after CR. This finding is in line with recent randomized controlled trials, including the use of applications and wearables to conventional CR [16–18]. Although the smartphone program incorporated several evidence-based behaviour-change techniques (i.e., goal-setting, personalised exercise prescriptions, action planning, prompting, and feedback) [10], these components alone may not be sufficient to meaningfully increase PA. Evidence from behaviour-change research indicates that digital prompts or nudges are most effective when embedded within a broader framework that includes regular, in-depth interactions with a healthcare professional [19]. In our trial, contacts occurred intermittently and were typically limited to brief, task-oriented exchanges through the in-app communication channel. Potentially, these interactions did not allow the degree of motivational coaching, barrier identification, problem-solving, or iterative goal adjustment that is considered essential for sustained behaviour change in mHealth-delivered CR [20]. So, a mHealth approach including several behaviour change techniques and repetitive bi-directional contact seems necessary to augment the effects of CR on PA.

Another explanation of the absence of the intervention effect is the high MVPA levels at baseline, i.e. participants performed almost 1 h of MVPA per day (i.e., 7 h/week). This amount of MVPA is almost 3 times higher than the WHO guidelines on PA (150 min/week of MVPA) [21] and seems already optimal for risk reduction of all-cause mortality [22]. The MVPA levels were also higher compared to a similar study population [6], and therefore, a ceiling effect may have reduced the overall intervention effect. The high PA levels at baseline may also be indicative of selection bias, as active and motivated individuals may have been more willing to participate in this study. Hence, the effect of our intervention on less active patients remains unknown and should be examined in future studies.

The absence of improvements in MVPA aligns with earlier studies reporting that MVPA is not or very weakly affected by contemporary CR programs [5, 23]. LIPA increased by 0.4 h/day in the control group, which was marginally higher compared to earlier studies (0.3 h/day) [5]. Additionally, daily step count increased by ~800 steps per day, which is likely clinically relevant as an increase in daily step count of around 500 steps was previously associated with a reduced risk for all-cause mortality and cardiovascular events in the general population [24]. Despite high PA metrics, our patients were also sedentary, as a high prevalence was observed for time spent sedentary > 9.5 h/day at baseline (78%) and post-CR (29%), which is associated with a higher risk of mortality [22]. The combination of high levels of MVPA and low sedentary time is most optimal for the prevention of adverse events [25], so reducing daily sedentary time offers room for further lifestyle improvements in this cohort.

### Strengths and limitations

Strengths of this study were the randomized controlled trial design, the concurrent center-based CR and smartphone training program approach, and objective assessments of PA. A limitation of this study was the concurrent timing of our intervention with the COVID-19 pandemic and its varying restrictions and lockdowns over time, which may have negatively impacted PA behavior and sitting time [26, 27]. Nevertheless, circumstances were equal between control and intervention groups, and the intervention primarily focused on increasing PA in the home environment (i.e., walking, cycling), which was not restricted during the lockdown phase. The COVID-19 lockdown likely induced a higher than anticipated drop-out rate (20%), but the a posteriori sample size calculation (75 participants) revealed that our study had sufficient power. Secondly, adherence to the smartphone program could not be extracted from the Virtual Training platform. This prevented analysis of engagement levels, which should be considered by future mHealth studies. Thirdly, no long-term follow-up measurements were performed. However, we do not expect long-term effects of the intervention as there were no effects directly post-CR, and earlier studies show that the effects of an intervention diminish over time [6, 28].

### Clinical implications

Adding a smartphone training program did not augment the effects of contemporary CR in this study, but more elaborate mHealth interventions still hold promise to make the transition of center-based to hybrid or home-based care. Important research gaps still exist about the optimal delivery, including the balance between centre-based and home-based components and the most effective technological modality [8], including the use of AI and large-language models. Future implementations may require clinician- or algorithm-assisted tailoring to enhance scalability and facilitate integration into routine clinical workflows. Furthermore, the timing of home-based mHealth support within the patient journey may warrant reconsideration. At the start of center-based CR, participants already participate in supervised-exercise training twice a week and are instructed by physiotherapists to improve habitual PA levels. Therefore, current guidance might already be sufficient in this rehabilitation phase. A home-based mHealth training program may be more valuable prior to CR as a preparation for center-based CR [29]. Moreover, maintaining higher PA levels following CR is challenging for CVD patients [6, 30]. Providing an mHealth maintenance program after CR seems, therefore, promising to sustain or improve PA following completion of CR [28].

## Conclusion

A smartphone training program on top of usual CR did not increase PA in patients with CAD. In addition, secondary outcomes, such as functional parameters, quality of life, and anxiety, were not impacted by the mHealth intervention. These findings highlight that a more elaborate mHealth intervention seems necessary to change habitual PA levels during CR in very active patients with CAD.

## Supplementary Information

**Supplemental Table S1.** CONSORT 2010 checklist

**Supplemental TableS2. **Descriptive statistics and constrained mixed model analysis outcomes

**Supplemental File S1. **Methods
