# Supplementary material for: A cardiac-rehab home-based mHealth program to improve physical activity in patients with coronary artery disease: a randomized controlled trial
Source: Neth Heart J. 2026 Apr 9;34(5):189–96. doi: 10.1007/s12471-026-02039-5 (PMC13090453; doi:10.1007/s12471-026-02039-5)
Supplement: Supplementary file 2 — Supplemental TableS2. Descriptive statistics and constrained mixed model analysis outcomes [file 12471_2026_2039_MOESM2_ESM.pdf]

Supplemental Table 2. Descriptive statistics and constrained mixed model analysis outcomes. Descriptive statistics at baseline and post-intervention are depicted for the total group, as well as for the intervention and control group separately. Control group was used as reference.

	Descriptive statistics<br><br>**Total**<br><br>**(n=93)**	**Intervention**<br><br>**(n=44)**	**Control**<br><br>**(n=49)**	Constrained mixed model<br><br>**Time*group interaction**<br><br>**(95% CI)**	**Cohen's d**<br><br>**(95% CI)**	**p-value interaction**
**Accelerometry**						
Moderate-to-vigorous intensity physical activity (h/day)						
Baseline (n=91)	0.8 [0.6-1.3]	0.8 [0.6-1.0]	0.9 [0.6-1.3]			
Post-CR (n=69)	0.9 [0.6-1.3]	0.9 [0.6-1.3]	1.0 [0.7-1.3]	-0.0 (-0.2; 0.1)	-0.1 (-0.4; 0.2)	0.57
Light intensity physical activity (h/day)						
Baseline (n=91)	3.6±1.3	3.9±1.4	3.4±1.3			
Post-CR (n=69)	4.1±1.4	4.2±1.5	3.9±1.3	0.1 (-0.4; 0.5)	0.0 (-0.3; 0.4)	0.79
Step count (steps/day)						
Baseline (n=91)	6,615 [4,652-9,249]	6,562 [5,081-9,998]	6,615 [4,571-9,226]			
Post-CR (n=69)	7,238 [5,347-9,127]	6,938 [5,047-9,186]	7,255 [5,516-9,127]	-227 (-1,277; 828)	-0.1 (-0.2; 0.2)	0.67
Sedentary time (h/day)						
Baseline (n=91)	10.8±1.6	10.5±1.6	11.0±1.7			

	Descriptive statistics<br><br>Total<br><br>(n=93)	Intervention<br><br>(n=44)	Control<br><br>(n=49)	Constrained mixed model<br><br>Time*group interaction<br><br>(95% CI)	Cohen's d<br><br>(95% CI)	p-value interaction
Post-CR (n=69)	10.3±1.7	10.2±1.8	10.5±1.5	-0.0 (-0.6; 0.5)	-0.0 (-0.3; 0.3)	0.95
**Functional parameters**						
Physical Fitness (Watt)						
Baseline (n=91)	89±29	88±25	90±32			
Post-CR (n=70)	94±27	89±21	98±32	-0 (-2; 2)	-0.0 (-0.1; 0.1)	0.95
Physical Fitness (RPE)						
Baseline (n=70)	14±1	14±2	14±1			
Post-CR (n=61)	13±1	13±1	13±1	-0.1 (-0.8; 0.5)	-0.1 (-0.6; 0.4)	0.72
Handgrip strength (kg)						
Baseline (n=92)	47±12	46±10	47±13			
Post-CR (n=71)	47±11	46±10	47±12	1.4 (-0.4; 3.2)	0.1 (-0.0; 0.3)	0.12
**HeartQoL and Cardiac Anxiety**						
HeartQoL total						

		Descriptive statistics<br><br>Total<br><br>(n=93)	Intervention<br><br>(n=44)	Control<br><br>(n=49)	Constrained mixed model<br><br>Time*group interaction<br><br>(95% CI)	Cohen's d<br><br>(95% CI)	p-value interaction
	Baseline (n=83)	1.5±0.6	1.5±0.6	1.6±0.6			
	Post-CR (n=60)	1.8±0.7	1.7±0.7	1.9±0.8	-0.1 (-0.4; 0.2)	-0.1 (-0.5; 0.3)	0.64
HeartQoL physical							
	Baseline (n=83)	1.4±0.7	1.3±0.7	1.5±0.7			
	Post-CR (n=60)	1.7±0.8	1.5±0.8	1.8±0.8	-0.1 (-0.5; 0.2)	-0.2 (-0.6; 0.3)	0.46
HeartQoL emotional							
	Baseline (n=83)	1.9±0.8	1.9±0.8	1.9±0.9			
	Post-CR (n=60)	2.0±0.9	2.1±0.8	2.0±0.9	-0.0 (-0.3; 0.3)	-0.0 (-0.4; 0.3)	0.81
Cardiac anxiety total							
	Baseline (n=84)	1.4±0.5	1.5±0.6	1.3±0.5			
	Post-CR (n=63)	1.2±0.5	1.3±0.5	1.1±0.5	0.1 (-0.1; 0.3)	0.2 (-0.1; 0.5)	0.20
Cardiac anxiety fear							
	Baseline (n=84)	1.6±0.7	1.6±0.7	1.5±0.7			

		Descriptive statistics<br><br>Total<br><br>(n=93)	Intervention<br><br>(n=44)	Control<br><br>(n=49)	Constrained mixed model<br><br>Time*group interaction<br><br>(95% CI)	Cohen's d<br><br>(95% CI)	p-value interaction
	Post-CR (n=63)	1.4±0.5	1.5±0.5	1.4±0.5	0.1 (-0.0; 0.3)	0.2 (-0.1; 0.5)	0.14
Cardiac anxiety avoidance							
	Baseline (n=84)	1.5±0.8	1.6±0.8	1.3±0.7			
	Post-CR (n=63)	1.1±0.8	1.2±0.8	1.0±0.7	0.1 (-0.2; 0.4)	0.1 (-0.3; 0.5)	0.57
Cardiac anxiety attention							
	Baseline (n=84)	1.0±0.6	1.1±0.7	0.9±0.6			
	Post-CR (n=63)	0.9±0.6	1.0±0.7	0.8±0.6	0.1 (-0.1; 0.3)	0.2 (-0.2; 0.5)	0.33

The control group is used as reference of the intervention outcomes. Descriptive statistics are presented as n (%) for categorical variables and as mean (± standard deviation) or median [interquartile range] for continuous variables. Constrained linear mixed model outcomes are presented as unstandardized beta (95% Confidence interval (CI)) for the interaction group*time.
