# Supplementary material for: A cardiac-rehab home-based mHealth program to improve physical activity in patients with coronary artery disease: a randomized controlled trial
Source: Neth Heart J. 2026 Apr 9;34(5):189–96. doi: 10.1007/s12471-026-02039-5 (PMC13090453; doi:10.1007/s12471-026-02039-5)
Supplement: Supplementary file 3 — Supplemental File S1. Methods [file 12471_2026_2039_MOESM3_ESM.docx]

**METHODS**

***STUDY SETTING AND POPULATION***

A parallel-group randomized controlled trial with two study arms was performed (Netherlands Trial Register: <https://onderzoekmetmensen.nl/nl/trial/21360>). The rationale and design of the Cardiac RehApp trial has previously been described in detail(1). There were no changes to the study protocol after trial commencement. Patients enrolled to CR at a large Dutch hospital (Canisius Wilhelmina Ziekenhuis, Nijmegen, the Netherlands), were screened for eligibility and informed by the specialized CR nurse. Inclusion criteria were 1) age >18 years, 2) a diagnosis of a coronary artery disease (i.e., ST-elevation myocardial infarction, non-ST-elevation myocardial infarction, unstable angina pectoris, or stable angina pectoris), 3) a referral to CR, 4) minimal digital skills to use a smartphone with the Virtual Training application, and 5) the ability to understand and perform the study procedures. Exclusion criteria were 1) no possession of a smartphone, or the inability to operate a smartphone (e.g., due to vision-, hearing-, cognitive- or dexterity impairment), 2) no (mobile) internet access at their place of residence, 3) contraindications to perform exercise during CR, 4) severe orthopedic problems that restrict physical activity, 5) significant language barriers. Informed consent was obtained from all patient prior to participation and the Cardiac RehApp trial protocol adhered to the ethical guidelines of the 1975 Declaration of Helsinki as reflected in a priori approval by the Medical Ethics Committee of the Radboud university medical center (NL72182.091.19). The CONSORT checklist is available in **Supplemental Table 1**.

***RANDOMIZATION***

Patients were enrolled, measured at baseline and, thereafter, randomly allocated (1:1) to the intervention or control group by the researcher. A computerized algorithm (Castor EDC 2021, Ciwit B.V., The Netherlands) was used for allocation concealment with random block sizes ranging from four to six patients. Randomization was stratified for index diagnosis (acute events *versus* elective procedures) to ensure balance of the treatment arms. Nurse specialists and patients were not blinded to the treatment allocation. Primary outcome analysis was blinded for the research team as an automatized script was used based on a unique participant identification number, independent from the randomization procedure.

***CENTER-BASED CR***

All patients received usual care and were seen by their cardiologist as clinically appropriate. Usual care consisted of a ~6-week comprehensive center-based CR program. Three regular consultations were scheduled with the nurse specialists focusing on lifestyle improvement, medication adherence and psychosocial well-being. Patients participated in an outpatient exercise program, consisting of a one hour group session of exercise training twice a week for 6 weeks supervised by a physiotherapist. Based on the individual patient’s needs, the standard CR program could be extended by a dietary module, psycho-educative prevention module and psychological module, or by additional consultations with the CR nurse.

***HOME-BASED TRAINING PROGRAM***

Participants in the intervention group received a 6-week smartphone training program alongside usual care CR. The participants were instructed to perform daily physical activities in their home situation using the Virtual Training® mHealth smartphone application (Welfaster ApS, Denmark)(2). The application contained different training programs including both strength and aerobic exercises (e.g., squats, walking, cycling) and provided synchornous instructions by video, text and audio. At baseline, the researcher and participant set individual goals based on preferences (e.g., biking or walking) and physical status (i.e., physical limitations, previous exercise experience, and age), which were then converted into a personalized home-based training program by the researcher. The smartphone program stimulated daily PA through goal-setting, personalised exercise prescriptions, and automated reminders in the home environment. Progression and feasibility were monitored via in-app messaging, and participants could contact the research team through the interactive platform when needed. These elements reflect common behaviour-change techniques such as action planning, prompting, and feedback, which are known to support increases in PA levels(3). The aerobic exercise program consisted of incremental walking or biking running with the goal to gradually increase exercise duration, intensity and distance. For resistance exercise, it was recommended to perform body weight exercises of 1-3 sets with 10-15 repetitions twice a week on a self-chosen moment. During CR, progression and feasibility of exercises were monitored with messages within the application, and automatic reminders were sent upon non-participation. Furthermore, an interactive platform was available in the Virtual Training® application to contact the research team.

***OUTCOME MEASURES***

All participants were assessed at baseline (prior to the CR program) and directly after completion of the 6-week CR program during a study visit to the hospital. The pre-specified primary outcome was change in objectively measured time spent in moderate-to-vigorous physical activity (MVPA), expressed in h/day, from baseline to directly post-CR (2). Secondary pre-specified outcomes included changes in sedentary time, physical fitness, handgrip strength, cardiovascular risk factors, quality of life and cardiac anxiety scores. Additionally changes in light intensity physical activity (LIPA) and step count were assessed.

***MEASUREMENTS***

***Accelerometery***

Physical activity patterns were objectively measured with a validated accelerometer (ActivPAL micro, PAL technologies, Glasgow, United Kingdom)(4, 5). The monitor was attached to the upper leg and continuously worn for 8 consecutive days. Furthermore, patients were requested to fill in a sleep diary to correct for sleeping time. Raw data was extracted using PAL Software Suite V.8 and analyzed by a modified version of the script of Winkler *et al*(6). Physical activity time was divided into moderate-to-vigorous physical activity (MVPA; activities with a MET value ≥3) and light physical activity (LIPA; i.e., activities with a MET-value <3) and were both expressed in h/day(7). Sedentary time was defined as any waking behavior characterized by an energy expenditure <1.5 MET while in seated, reclined or lying posture(8, 9). Step count was expressed as steps/day. Measurements were performed at baseline (prior to the CR program) and directly after completion of the 6-week CR program

***Functional parameters***

The Åstrand test (i.e., a submaximal cycling test) was performed to examine physical fitness. In short, subjects performed a 6-minute cycling test on a constant workload that ensures a stable heart rate between 110 and 140 bpm(1). During the test, heart rate was continuously monitored (Polar V800, Kempele, Finland), and the Borg score was reported during the third and sixth minute. Patients using heart rate lowering medication (e.g., betablockers) followed an adjusted test in which the workload was steadily increased similar to the standard test until a Borg Rating of Perceived Exertion (RPE) score of at least 12 was reached. The change in workload and RPE from pre- to post-CR was used for analysis to estimate changes in physical fitness. Hand grip strength was assessed in the dominant hand using a hydraulic, analogue hand dynamometer (Jamar, Jackson, MI, USA). Three measurements were performed, and the maximum strength effort (kg) was used for analysis.

***Quality of life and cardiac anxiety***

Quality of life was measured using the HeartQoL, which is a validated, 14-item questionnaire that can be converted into a total, physical and emotional score (range from 0 (poor) to 3 (better))(10). Cardiac anxiety was measured using the 18-item, validated Cardiac Anxiety Questionnaire (CAQ)(11). Total scores, and domain scores for fear, avoidance and attention were calculated, in which a higher score reflects a higher cardiac anxiety (range from 0 (never) to 4 (always))(11).

***SAMPLE SIZE***

The described study is part of a larger trial of which a sample size calculation was performed prior to trial initialization and described previously(2). A posteriori, the sample size needed for our research question was calculated. As originally described, a change in the primary outcome MVPA of 20 min/day was considered reasonable and significant clinical relevant(2). Considering a standard deviation of 23 min/day, the calculated effect size was 0.33 and an alpha of 0.05 and beta of 0.2 (power 0.8) were used(2). According to G*power (version 3.1.9.4, Kiel University, Germany)(12), a total of 75 participants should be included to answer this research question comparing the intervention and control group (**Figure 1**).


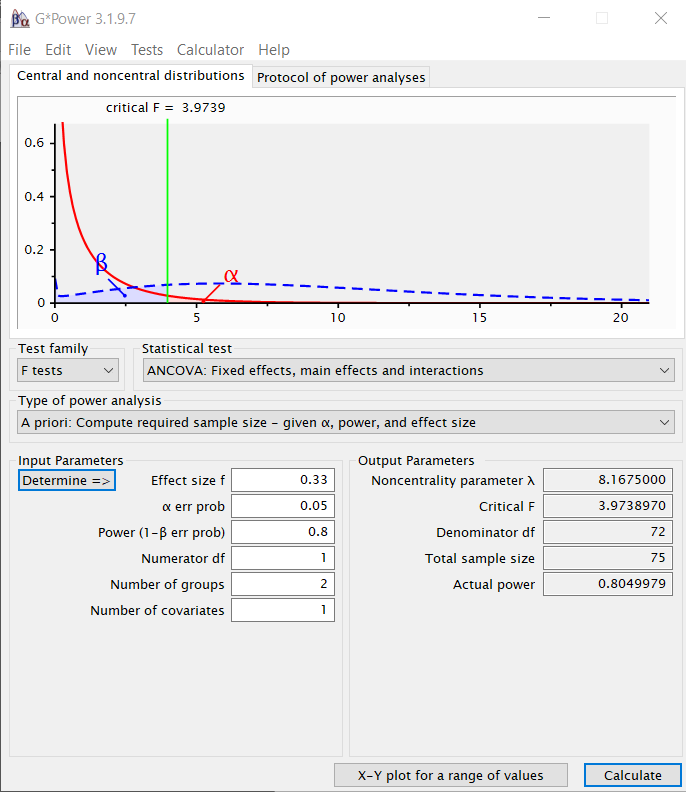


**Figure 1.** Sample size calculation.

***STATISTICAL ANALYSIS***

All statistical analyses were performed using R version 4.2.1 with packages “lme4” and “Lmmstar” (visualization purposes), and “emmeans”. All tests were two-sided, confidence intervals (CI) were at the 95% level and P-values <0.05 were considered statistically significant. Continuous normally distributed data were presented as mean ± standard deviation (SD), continuous not-normally distributed data as median [interquartile range] (IQR) and categorical variables as number (%). All data were visually inspected for normality.

Primary and secondary outcome analysis were performed on a modified intention-to-treat basis including all participants with at least one primary outcome measurement at baseline or post-CR. A baseline adjusted (i.e constrained) linear mixed-model analysis was used to handle missing data and to avoid baseline imbalances between treatment arms(13). Time (categorical) and the interaction time*group were included in the model.

**References**

1. Vonk T, Bakker EA, Zegers ES, Hopman MTE, Eijsvogels TMH. Effect of a personalised mHealth home-based training application on physical activity levels during and after centre-based cardiac rehabilitation: rationale and design of the Cardiac RehApp randomised control trial. BMJ Open Sport Exerc Med. 2021;7(3):e001159.

2. Vonk T, Bakker EA, Zegers ES, Hopman MTE, Eijsvogels TMH. Effect of a personalised mHealth home-based training application on physical activity levels during and after centre-based cardiac rehabilitation: rationale and design of the Cardiac RehApp randomised control trial. BMJ Open Sport & Exercise Medicine. 2021;7(3):e001159.

3. Patterson K, Davey R, Keegan R, Freene N. Smartphone applications for physical activity and sedentary behaviour change in people with cardiovascular disease: A systematic review and meta-analysis. PLoS One. 2021;16(10):e0258460.

4. Harrington DM, Welk GJ, Donnelly AE. Validation of MET estimates and step measurement using the ActivPAL physical activity logger. J Sports Sci. 2011;29(6):627-33.

5. Kozey-Keadle S, Libertine A, Lyden K, Staudenmayer J, Freedson PS. Validation of wearable monitors for assessing sedentary behavior. Med Sci Sports Exerc. 2011;43(8):1561-7.

6. Winkler EA, Bodicoat DH, Healy GN, Bakrania K, Yates T, Owen N, et al. Identifying adults' valid waking wear time by automated estimation in activPAL data collected with a 24 h wear protocol. Physiol Meas. 2016;37(10):1653-68.

7. Lyden K, Keadle SK, Staudenmayer J, Freedson PS. The activPALTM Accurately Classifies Activity Intensity Categories in Healthy Adults. Med Sci Sports Exerc. 2017;49(5):1022-8.

8. Eckel RH, Jakicic JM, Ard JD, de Jesus JM, Houston Miller N, Hubbard VS, et al. 2013 AHA/ACC guideline on lifestyle management to reduce cardiovascular risk: a report of the American College of Cardiology/American Heart Association Task Force on Practice Guidelines. J Am Coll Cardiol. 2014;63(25 Pt B):2960-84.

9. Tremblay MS, Aubert S, Barnes JD, Saunders TJ, Carson V, Latimer-Cheung AE, et al. Sedentary Behavior Research Network (SBRN) - Terminology Consensus Project process and outcome. Int J Behav Nutr Phys Act. 2017;14(1):75.

10. De Smedt D, Clays E, Hofer S, Oldridge N, Kotseva K, Maggioni AP, et al. Validity and reliability of the HeartQoL questionnaire in a large sample of stable coronary patients: The EUROASPIRE IV Study of the European Society of Cardiology. Eur J Prev Cardiol. 2016;23(7):714-21.

11. van Beek MH, Voshaar RC, van Deelen FM, van Balkom AJ, Pop G, Speckens AE. The cardiac anxiety questionnaire: cross-validation among cardiac inpatients. Int J Psychiatry Med. 2012;43(4):349-64.

12. Faul F, Erdfelder E, Lang AG, Buchner A. G*Power 3: a flexible statistical power analysis program for the social, behavioral, and biomedical sciences. Behav Res Methods. 2007;39(2):175-91.

13. Coffman CJ, Edelman D, Woolson RF. To condition or not condition? Analysing 'change' in longitudinal randomised controlled trials. BMJ Open. 2016;6(12):e013096.
